# Attentional Switches and Dual-Task Interference

**DOI:** 10.1371/journal.pone.0118216

**Published:** 2015-03-02

**Authors:** Janne F. Ettwig, Adelbert W. Bronkhorst

**Affiliations:** 1 Vrije Universiteit, Amsterdam, The Netherlands; 2 TNO, Soesterberg, The Netherlands; University of Groningen, NETHERLANDS

## Abstract

In four experiments, we studied the time course of interference between detection of an oddball orientation target (OT) in an 8-item circular search display, and identification of a letter target (LT) in a central stream of distractor letters. Dual-task performance for different temporal lags between targets was compared to single-task performance. When the LT preceded the OT, dual-task performance levels were reduced at short inter-target intervals of 0 and 166 ms; when the OT preceded the LT, the dual-task interference was unexpectedly stronger and lasted for up to 500 ms. Resource competition due to temporally overlapping target processing cannot account for this result, because the feature search task is easier than the letter identification task, and therefore would have generated less interference when presented first. Two alternative explanations were explored. First, by manipulating the spatial inter-target distance, we investigated to what degree there is a penalty associated with directing the attentional window from a large object (the search display) to a smaller object (the central letter stream). Second, by varying the duration of the OT and subsequent mask, we studied whether the interference was caused by the difficulty of disengaging attention from the search display. Results support this second explanation and thus indicate that switching attention to the letter stream is hampered by the continuing presence of (masked) search display items. This result shows that attentional effects may play a major role in dual-task execution and can easily obscure interference due to other factors such as resource competition.

## Introduction

Everyday experience teaches us that it can be very difficult to perform several tasks at once. Multitasking is possible (e.g. [1]), but when both tasks are visual, performance is often degraded (e.g. [2]). Think for example of trying to find your way on a map while watching the road. Our brain has to deal with similar inputs simultaneously which can lead to interference [3,4]. The question how we prioritize the most relevant information from different sources has been the subject of extensive research (e.g. [5]).

Previous research posits that performing two visual tasks at the same time is nearly impossible without performance penalties, even if one of the two tasks is relatively easy [3–5]. Joseph, Chun and Nakayama [3] had participants search for a pop-out orientation target and compared performance to the dual task condition where the identification of a letter target in a Rapid Serial Visual Presentation (RSVP) task was added. They not only found strongly reduced dual-task performance for short inter-target intervals, but also that the reduction persisted at relatively long intervals (up to around 350 ms). This result is particularly interesting because pop-out stimuli can be easily detected, irrespective of the number of surrounding distracters [6], which suggests that they are processed preattentively, requiring little or no resources (e.g. [7–10]). Thus, it was expected that a pop-out search would not interfere with another task, even one as demanding and resource consuming as an RSVP task [11]. Joseph and colleagues, therefore, concluded that the notion of unlimited capacity for perception of pop-out stimuli was not supported by their results. Instead, it seems that even processing such stimuli suffers from an attentional bottleneck and limited processing resources.

However, recent results of Ettwig and Bronkhorst [12] indicate that this conclusion needs to be modified. These authors showed that when the orientation search task used by Joseph and colleagues is replaced by a color- or motion-search task, the interference with a letter-RSVP task disappears completely. They also found that the interference re-emerged when the saliency of the search targets was reduced, so that single-task performance was the same for all types of targets, but only at lag 0 ms, i.e. when the RSVP and search targets were presented simultaneously. Thus, while supporting the notion that pop-out search is not free from interference with other task, the results of Ettwig and Bronkhorst [12] further demonstrate that the degree and duration of the interference depends strongly on both task difficulty and the specific combination of target features.

Why is it then that the combination of a letter-RSVP with an orientation search display yields such a strong and lasting interference, even exceeding the duration required for a spatial shift of attention [13]? The most probable explanation is that it is due to competition for higher-level processing resources required by both tasks [14–20]. The underlying assumption is that processing of the first target takes a relatively long time, so that it is still ongoing when the second target reaches the processing stage. This is consistent with the relative difficulty of the RSVP task, which involves discrimination of a target in a letter stream presented with a high (>10/s) rate [11]. A direct test of this explanation would, therefore, be to determine whether replacement of the first task by one that is less difficult results in less dual-task interference. The only straightforward way to do this without changing the combination of tasks, is by reversing the task order. Because the search task is relatively easy, the prediction would be that when it precedes the RSVP-task, overlap in the processing of the two target stimuli should only occur at very short inter target intervals in the dual-task condition.

We tested this prediction by performing a replication of the dual-task experiment of Joseph, Chun and Nakayama [3], in which the RSVP-target precedes the orientation target, followed by a second experiment in which the task order was reversed. While the first experiment corroborated the dual-task interference observed earlier [3,12], the second experiment failed to confirm the prediction of decreased interference. The subsequent experiments described in this paper were designed to test two alternative hypotheses explaining this unexpected finding. Both hypotheses presume that the dual-task interference observed in the second experiment is caused by (early) attentional effects and not by competition for (higher-level) processing resources.

The first hypothesis is based on the dynamic properties of the attentional window. Previous studies have demonstrated that looming stimuli capture attention more readily than receding stimuli do [21–24]. The explanation might be one of biological urgency. An approaching stimulus is potentially more relevant for survival and therefore strongly attracts attention, especially when on a collision path with the observer [25]. Similarly, experiments by Takeuchi [26] show, that expanding gratings amid receding distractors are more easily detected than vice versa. We, therefore, hypothesize that observers switch more easily from a small attentional window (encompassing the RSVP stimuli) to a large window (also including the surrounding search display) than vice versa and that this asymmetry causes the larger than predicted interference found for Experiment 2.

The second hypothesis is based on attentional capture and the time course of attentional switching. As reviewed by Posner and Petersen [27], three separate stages can be distinguished in a switch of attention: disengagement, shifting and engagement. This is illustrated in the gap or overlap paradigm. When reacting to a peripherally presented target, the presence of a central fixation dot (overlap condition) leads to delayed reaction times, compared to conditions where the central fixation is removed shortly before the peripheral target is presented (gap condition) (e.g. [28]). It is hypothesized that disengagement of attention is impeded by the presence of the central fixation dot, but is facilitated by its removal, allowing participants to deploy attention to the peripheral target more rapidly [28–30]. Although the present search display is not presented centrally and is therefore not directly comparable to the fixation used in the gap overlap paradigm, its size probably compensates for the larger neural representation of stimuli presented more foveally, e.g. [31]. The second hypothesis, thus, poses that the mere presence of the search display and subsequent mask captures and holds attention, preventing it from disengaging even after the search stimuli have been perceived. Only when the mask is removed, attention is released and can then shift to the RSVP stream.

## Experiment 1 – Interference between a Search Task and a Preceding Letter-Discrimination Task

### Methods

**Ethics statement.** The present and all following experiments, including the consent procedure, were approved by the ethics board of the Faculty of Psychology and Education (VCWE) and conducted according to the principles of the Declaration of Helsinki. Participants received information about the study and their rights and gave a digital informed consent. The study was not associated with any risks for participants (it was non-invasive) and all data obtained during this study were analyzed anonymously.

**Participants.** 18 students (7 male; 1 left-handed; mean age 24 years; age range 18 to 29 years) participated for course credits or money. All had normal or corrected-to-normal vision.

**Fig 1:**
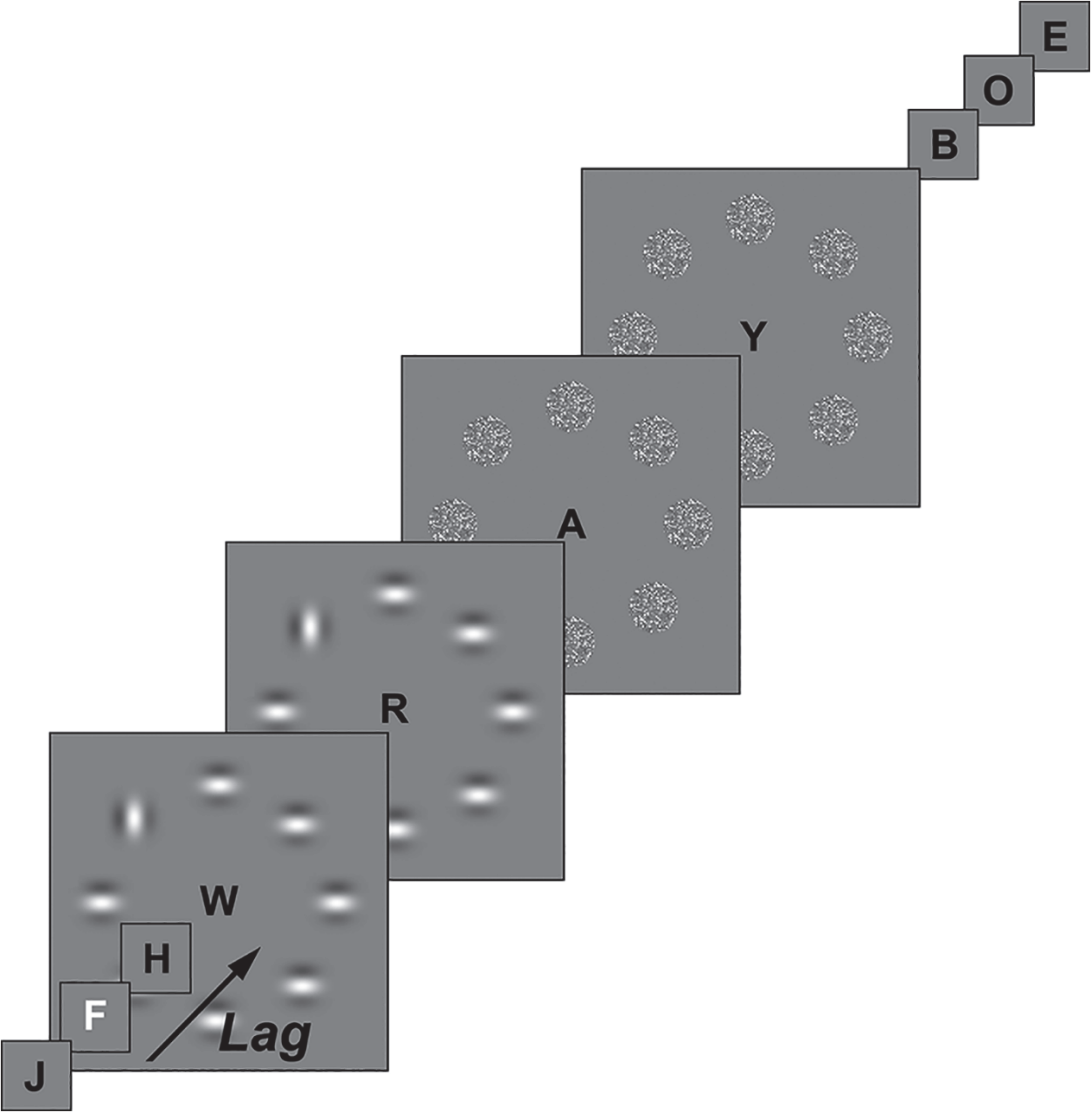
Timeline of Experiment 1. Every letter is presented for 83 ms, followed by a 50 ms blank. The OT search screen and the consecutive mask are presented for 166 ms.

**Stimuli & apparatus.** Participants were seated in a dimly lit room looking at the visual stimuli that were displayed on a 22-inch LCD monitor, on a gray background (25 cd/m2). The participants viewed the monitor binocularly from a 70 cm-distance with their head placed in a headrest. In this dual-task experiment, the first task consisted of the identification of a white letter target (LT) in an RSVP stream of black letters. The start of the RSVP stream always marked trial onset. The RSVP stream consisted of randomly (without replacement) chosen black, uppercase, letters, “J” and “N” excluded (0 cd/m2; 36 arcmin tall; font Geneva). The LT was a white, uppercase, letter (96.5 cd/m2; 36 arcmin tall) randomly chosen from the same set as the non-targets and could be displayed at the sixth to eleventh position in the RSVP stream. The LT was present in every trial. Each letter was presented for 33 ms followed by a 50 ms blank interval; see Fig. 1. The second task comprised detecting a differently oriented Gabor patch, the orientation target (OT), in-between 7 homogenously oriented distractor patches. The Gabor patches were constructed with a Gaussian envelope of 50% peak contrast and a standard deviation of 22 arcmin. They had a cosine modulation of 110 arcmin wavelength and were either horizontally or vertically oriented. The Gabor patches were regularly spaced on an imaginary circle at 5.3° eccentricity. A target in the form of a uniquely oriented Gabor was present in 50% of the trials. The Gabor patches were presented for 166 ms and were followed by a mask for the same duration, while the RSVP stream continued. The mask was generated by pixilating and scrambling the Gabor patches. Each trial was terminated after 415 ms after the last target presentation.

**Procedure & design.** Participants performed a single-task condition in which they identified the OT but ignored the LT and a dual-task condition in which they both identified the LT and detected the OT. The participants were instructed to maintain fixation on the RSVP stream at all times during the experiment. For practice, participants first viewed screenshots of the search display and then performed 10 trials of each condition, the first half with and the second half without feedback. During the experiment itself no feedback was given. Each trial started with the presentation of a fixation dot for 500 ms followed by a 500 ms blank screen after which the RSVP stream started. The LT was presented first, and after one of five different lags (0, 166, 498, 747, 996 ms) the OT followed. Note that a lag of 0 ms implies simultaneous onset of both the ring of Gabor patches and LT.

After each trial, participants gave an unspeeded response to OT detection, pressing the “J” key when they did detect OT or the “N” key when they did not. In the dual-task condition, they additionally responded to the LT by pressing the corresponding letter on a QWERTY keyboard. The experiment consisted of 6 alternating blocks of single- and dual-task conditions, each comprising 60 trials. The starting condition was counterbalanced across participants. Within the blocks LT position, OT orientation and location, and LT identity were randomized across trials, and OT presence and lags were counterbalanced.

### Results and Discussion

In all experiments, OT accuracy is based on correct performance, i.e. using both trials in which OT presence was correctly detected and correctly rejected. Furthermore, dual-task performance data for OT detection are analyzed given correct LT identification. Because of this, the number of available trials per lag varies slightly (between 22 and 25 for the current experiment). Furthermore, subjects who, during the experiment, achieved less than 10 data points in any one condition were excluded from the experiment. In the current experiment, this was the case for two participants.

**Fig 2:**
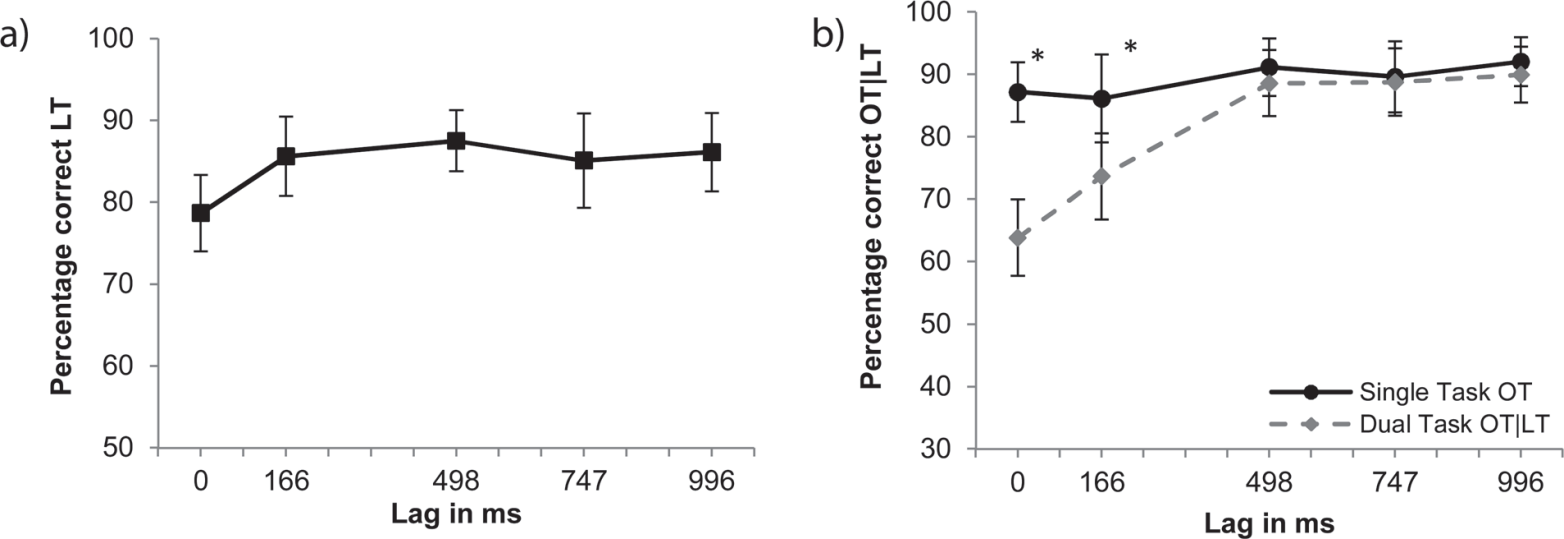
Results of Experiment 1. Panel a: Dual-task letter identification (LT). Panel b: Single- and dual-task performance given correct LT for orientation detection (OT). The solid line represents the single-task condition. Stars mark significant differences between single- and dual-task conditions (*p* < 0.007).

**LT identification.** The data for LT identification (see Fig. 2A) show that performance averaged at 84.6% correct in the dual-task condition. To evaluate the difference between consecutive lags, four Bonferroni-corrected paired samples t-tests (α = 0.013) were performed. They reveal a significant difference in performance between lags 0 ms and 166 ms [*p* < 0.009].

**OT detection.** The data for OT detection (see Fig. 2B) show that performance averaged at 89.2% correct for the single-task condition versus 80.9% correct for the dual-task condition. An analysis of variance (ANOVA) on this data revealed an effect of condition [*F*(1, 15) = 19.94, *MSE* = 136.92, *p* < 0.001] and lag [*F*(4,60) = 34.08, *MSE* = 45.44, *p* < 0.001], as well as an interaction between condition and lag [*F*(4, 60) = 14.8, *MSE* = 50.15, *p* < 0.001]. Five Bonferroni-corrected paired-samples t-tests (α = 0.01) showed that T2-detection in the single condition differed for lags 0 and 166 ms from the dual condition (*p* < 0.007).

Results show that performance for the single task is independent of lags but that dual-task performance is reduced also for nonzero lags, as found earlier by Joseph et al. and Ettwig and Bronkhorst [3,12]. An explanation for the dual-task interference is that, because of the difficulty of the RSVP task, the LT is still being processed when the OT arrives, resulting in competition for processing resources needed by both tasks. In the next experiment, we test this explanation by reversing the order of the tasks. Presenting the (easy) search task first while keeping the set of tasks the same, presumably leads to dual-task interference with a shorter duration.

## Experiment 2 – Interference between Letter Discrimination and a Preceding Search Task

### Methods

**Participants.** 14 students (4 male; 1 left-handed; mean age 21.4 years; age range 18 to 26 years) participated for course credits or money. All had normal or corrected-to-normal vision.

**Stimuli & apparatus.** Apart from the following changes, Experiment 2 was the same as Experiment 1. The single task condition now consisted of identification of the LT, the dual task was again the combination of detecting the OT as well as identifying the LT. Importantly, in the current experiment the OT was always presented first and was followed by the LT. OT onset was either 415, 498 or 581 ms after start of the RSVP stream and thus the trial.

### Results and Discussion

Dual-task performance data for LT identification are analyzed given correct OT detection. Because of this, the number of available trials per lag is not constant, but varies slightly (between 26 and 29 for the current experiment).

**Fig 3:**
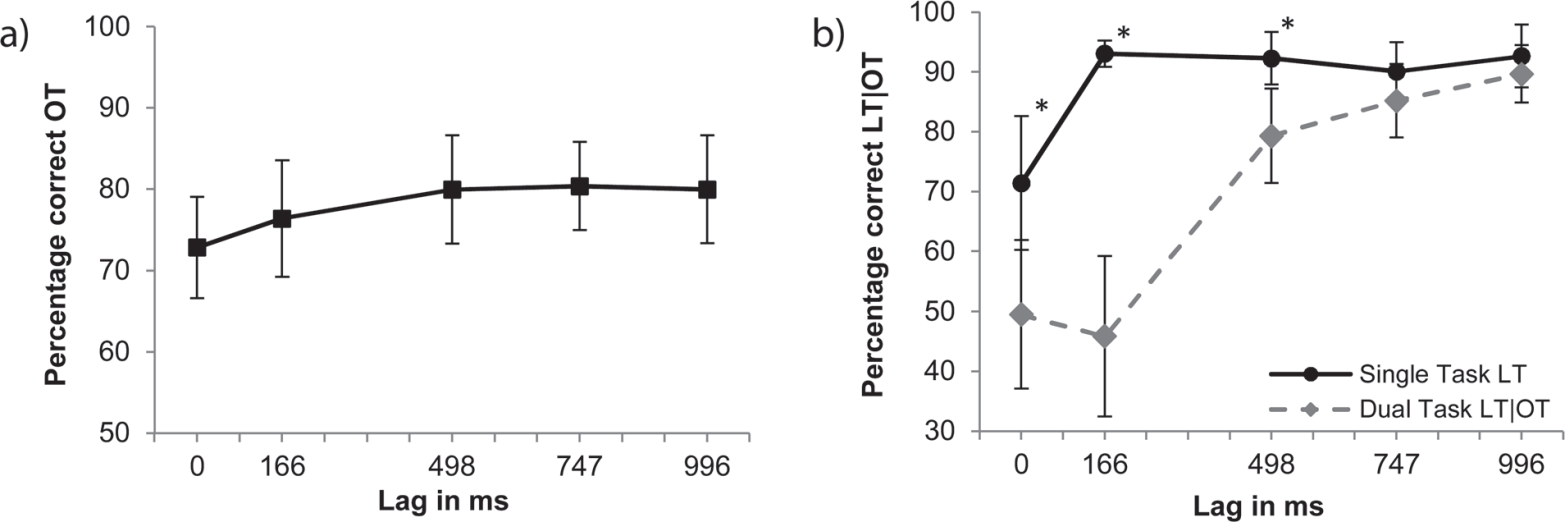
Results of Experiment 2. Panel a: Dual-task Gabor detection performance (OT). Panel b: Single- and dual-task performance given correct OT for letter identification (LT). The solid line represents the single-task condition. Stars mark significant differences between single- and dual-task conditions (*p* < 0.001).

**OT detection.** Orientation-target detection averaged at 77.8% of correctly detected targets over all lags. To compare performance for consecutive lags, four Bonferroni-corrected paired samples t-tests (α = 0.013) were performed. No significant performance difference were detected (*p* > 0.18), see Fig. 3A.

**LT identification.** The data for LT identification (see Fig. 3B) indicate that performance in the single-task condition was better than in the dual-task condition, especially for earlier lags. Performance averaged at 88% correct for the single-task versus 69.4% correct for the dual-task condition. To examine this difference more closely, a 2x5 ANOVA with condition (single/dual task) and lag (0, 166, 498, 747 and 996 ms) as factors was performed. This revealed main effects for condition [*F*(1,13) = 61.6, *MSE* = 184, *p* < 0.001] and lag [*F*(4,52) = 20.8, *MSE* = 236.6, *p* < 0.001], and an interaction between condition and lag [*F*(4,52) = 18.8, *MSE* = 120.4, *p* < 0.001]. Five Bonferroni-corrected paired-samples t-tests (α = 0.01) showed significant differences between single- and dual-task performances at lags 0, 166 and 498 ms (*p* < 0.001), but not at larger lags (*p* > 0.18).

Single-task identification of the target letter showed a clear dip in performance at lag 0 ms. Although this effect is similar to that observed for dual-task LT identification in Experiment 1, the crucial difference is that here, no relevant information can be extracted from the secondary task. It, thus, seems that the abrupt onset of the ring of Gabor patches still captures attention, even if it is irrelevant for the task at hand [13,32–36].

Dual task performance shows deep and prolonged interference. Notable is that the difference is largest at lags 0 (21.9%) and 166 ms (47.2%) but still significant at 498 ms (13%). We analyzed performance difference between the two different target orders that are tested in Experiment 1 and 2 with an 2x2x5 ANOVA. Target order (LT before OT, OT before LT), condition (single/dual task) and lag (0, 166, 498, 747 and 996 ms) were used as factors. This revealed a significant difference between the single task conditions for lag 0 ms [*F*(1,28) = 7.0, *p* < 0.013, *r* = 0.35] as well as between the dual task conditions for lags 0 ms [*F*(1,28) = 4.4, *p* < 0.044, *r* = 0.31] and 166 ms [*F*(1,28) = 14.1, *p* < 0.001, *r* = 0.39]. In all instanced, performance in Experiment 1 was higher than in Experiment 2. Thus, contrary to our expectations, dual-task interference is more profound in the current experiment than when the LT precedes the OT [37–41].

As discussed in the Introduction, a possible explanation for this result might be found in a spatial asymmetry in directing attentional focus. Previous research demonstrates that looming targets are more easily picked up then receding stimuli [21–24], which suggests that it is easier to spatially expand the attentional window than to constrict it. Because the search display covers a much larger area than the RSVP stream, this difference might, thus, have caused the relatively poor dual-task performance observed in Experiment 2. The following experiment was designed to examine whether changes in the size of the attentional window indeed affect dual-task performance. We again presented the OT before the LT but manipulated the radius of the OT search display. It was either smaller than that used in the previous experiments, or about twice as large. The hypothesis is that dual-task performance reduces as a function of search display size.

## Experiment 3 – Varying the Radius of the Search Display

In this experiment the spatial distance between the search display and the RSVP stream is varied to assess whether this affects dual task interference between an LT and a preceding OT.

### Methods

**Participants.** 18 students (2 male; 2 left-handed; mean age 21 years; age range 18 to 28 years) participated for course credits or money. All had normal or corrected-to-normal vision.

**Stimuli & apparatus.** Apart from the following changes, Experiment 3 was the same as Experiment 2. The Gabor Patches were now presented on two different imaginary circles, one with a 4°- and one with a 10.8°-radius around the central letter stream. Furthermore the number of lags was reduced from five to three: 0, 166 and 747 ms to limit experiment duration.

### Results and Discussion

As in the first experiment, we observed only small differences in the number of trials available per lag in the dual-task condition, ranging from 17 to 19 trials. Subjects who, during the experiment, achieved less than 10 data points in any one condition were excluded from the experiment. This was the case for three participants.

**Fig 4:**
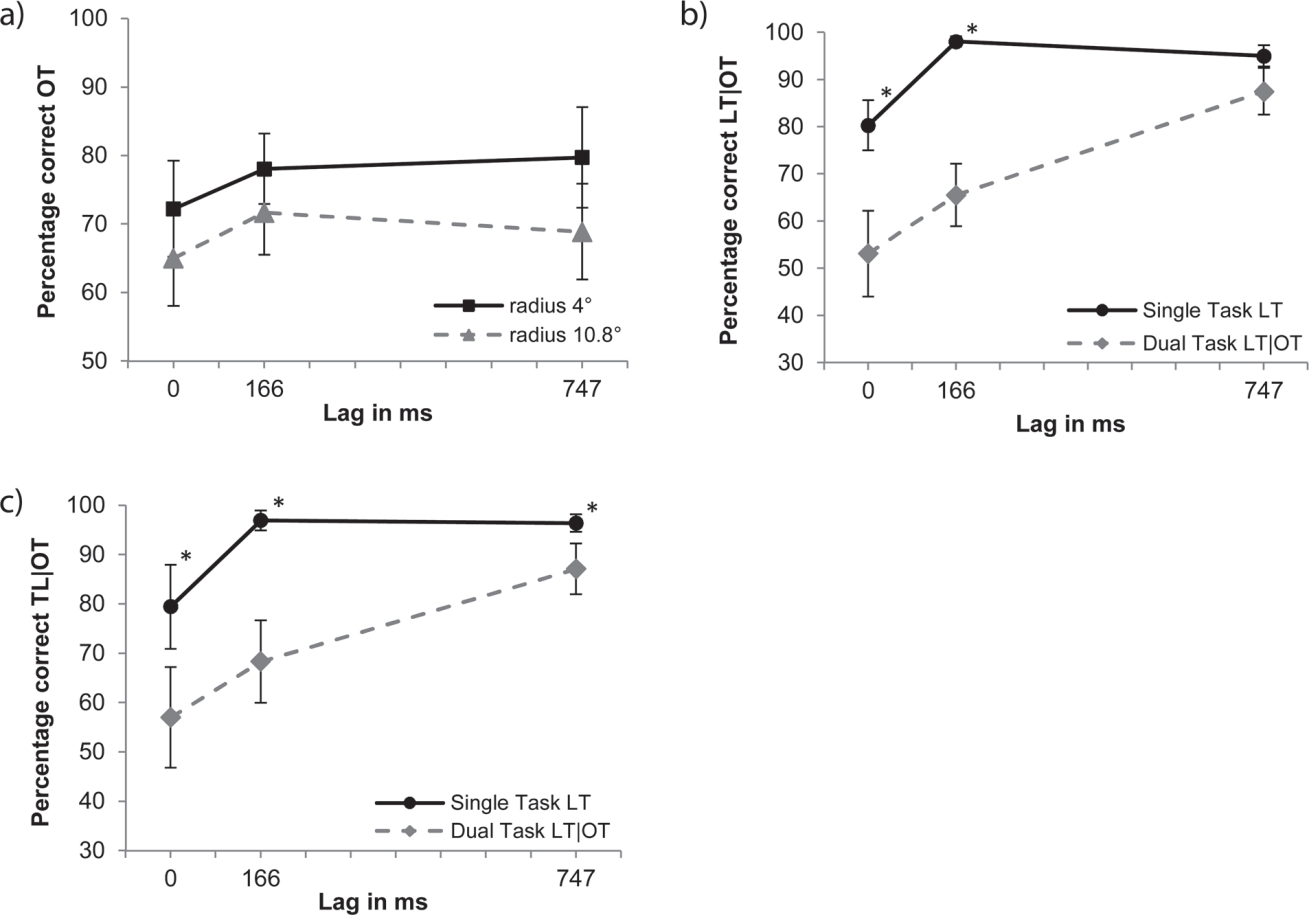
Results of Experiment 3. Panel a: Dual task Gabor detection performance (OT). The solid line represents performance for the 4° radius, the dashed line represents 10.8°. Panels b and c: Single- and dual-task performance given correct OT for letter identification (LT) for radii 4° and 10.8°, respectively. The solid lines represents the single-task condition. Stars mark significant differences between single- and dual-task results (*p* < 0.006).

**OT detection.** To evaluate orientation-target detection performance for different lags, a 2x3 ANOVA was performed with radius (4°, 10.8°) and lag (0, 166, 747 ms) as factors. Effects of radius [*F*(1,14) = 7, *MSE* = 212.8, *p* < 0.019] and lag [*F*(1,14) = 3.8, *MSE* = 94.4, *p* < 0.035] were found. Subsequently, three Bonferroni-corrected paired samples t-tests (α = 0.017) were performed to determine the difference between lags averaged over radii, however, no difference was found (*p* > 0.03), see Fig. 4A.

**LT identification.** LT identification scores for radius 4° (see Fig. 3, Panel b) and for radius 10.8° (Panel c) show that performance in the dual-task condition is generally lower than in the single-task condition. To evaluate this statistically, a 2x2x3 ANOVA with radius (4° and 10.8°), condition (single/dual task), and lag (0, 166 and 747 ms) as factors was performed. Main effects of condition [*F*(1,14) = 122, *MSE* = 166.8, *p* < 0.001] and lag [*F*(2,28) = 37.1, *MSE* = 237.7, *p* < 0.001], as well as an interaction between condition and lag [*F*(2,28) = 14, *MSE* = 141.6, *p* < 0.001] were found. However neither the main effect of radius nor any interaction of radius with other factors (including the 3-way interaction) showed significance.

These results show that manipulating the radius of the search display has no effect on dual-task interference. This falsifies our first hypothesis, which claims that dynamic changes in size of the attentional window explain the large interference found in Experiment 2. In the following experiment, we therefore investigate the second hypothesis discussed in the Introduction, which poses that the interference found in Experiment 2 is due to difficulties in disengaging attention from the orientation-search display, even after the relevant information is replaced by a mask. Earlier research involving the gap overlap paradigm suggests that the continuous presence of a stimulus that holds or held relevance for the task impedes disengagement of attention [28–30]. If attention dwells upon the search display even after the target is removed, dual-task interference will depend on the presentation durations of target and mask.

## Experiment 4 – Varying the Durations of Targets and Masks in the Search Display

In this experiment the OT presentation duration as well as the combined target/mask duration of the OT are manipulated to examine its effect on dual-task interference.

### Methods

**Participants.** 20 students (3 male; 3 left-handed; mean age 21 years; age range 18 to 27 years) participated for course credits or money. All had normal or corrected-to-normal vision.

**Fig 5:**
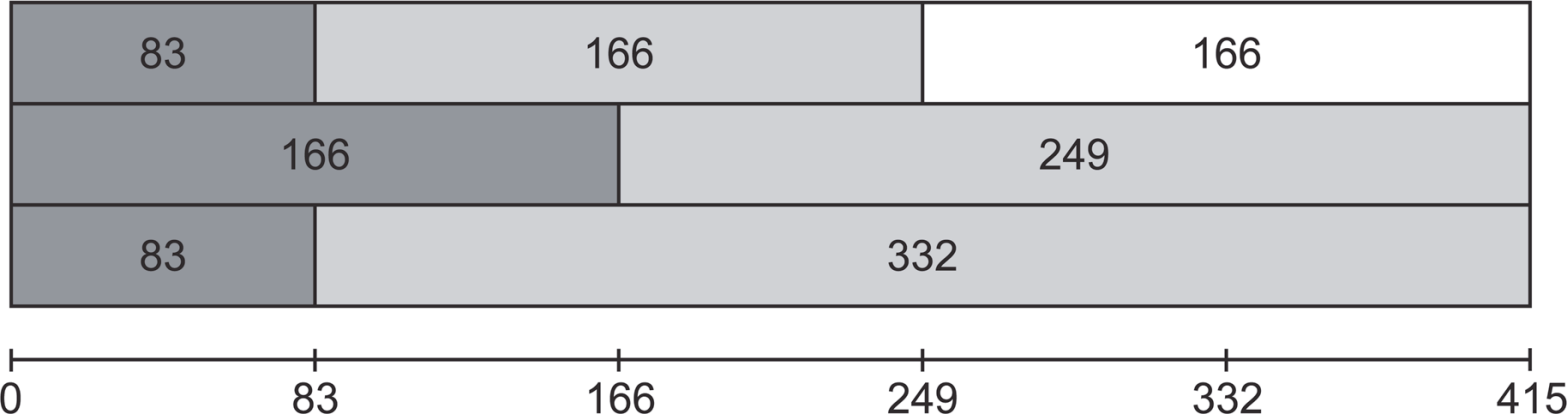
Timeline of target- and mask-presentation duration combinations.

**Stimuli & apparatus.** Apart from the following changes, Experiment 4 was the same as Experiment 2. Instead of presenting both the OT and the consecutive mask for 166 ms, the following three combinations of OT- and mask-durations were used: 83 ms and 166 ms, 166 ms and 249 ms, and 83 ms and 332 ms. Thus, there were two different OT durations (83 and 166 ms) and two different combined durations (249 ms and 415 ms) for an overview, see Fig. 5.

All durations are multiples of the presentation duration of the letters in the RSVP stream. The particular combinations were chosen to determine whether results depend only on target duration (in that case the 83/166 ms and 83/332 ms combinations would yield the same results) or on the combined target + mask duration (then, the 166/249 ms and 83/332 ms combinations would yield the same results). In order to limit the number of conditions and still collect sufficient data in the region where dual-task interference was expected, lags of 166, 415 and 581 ms were used in this experiment.

### Results and Discussion

All lags had identical trial counts. Four subjects achieved less than 10 data points in any one cell during the experiment and were therefore excluded from the experiment.

**Fig 6:**
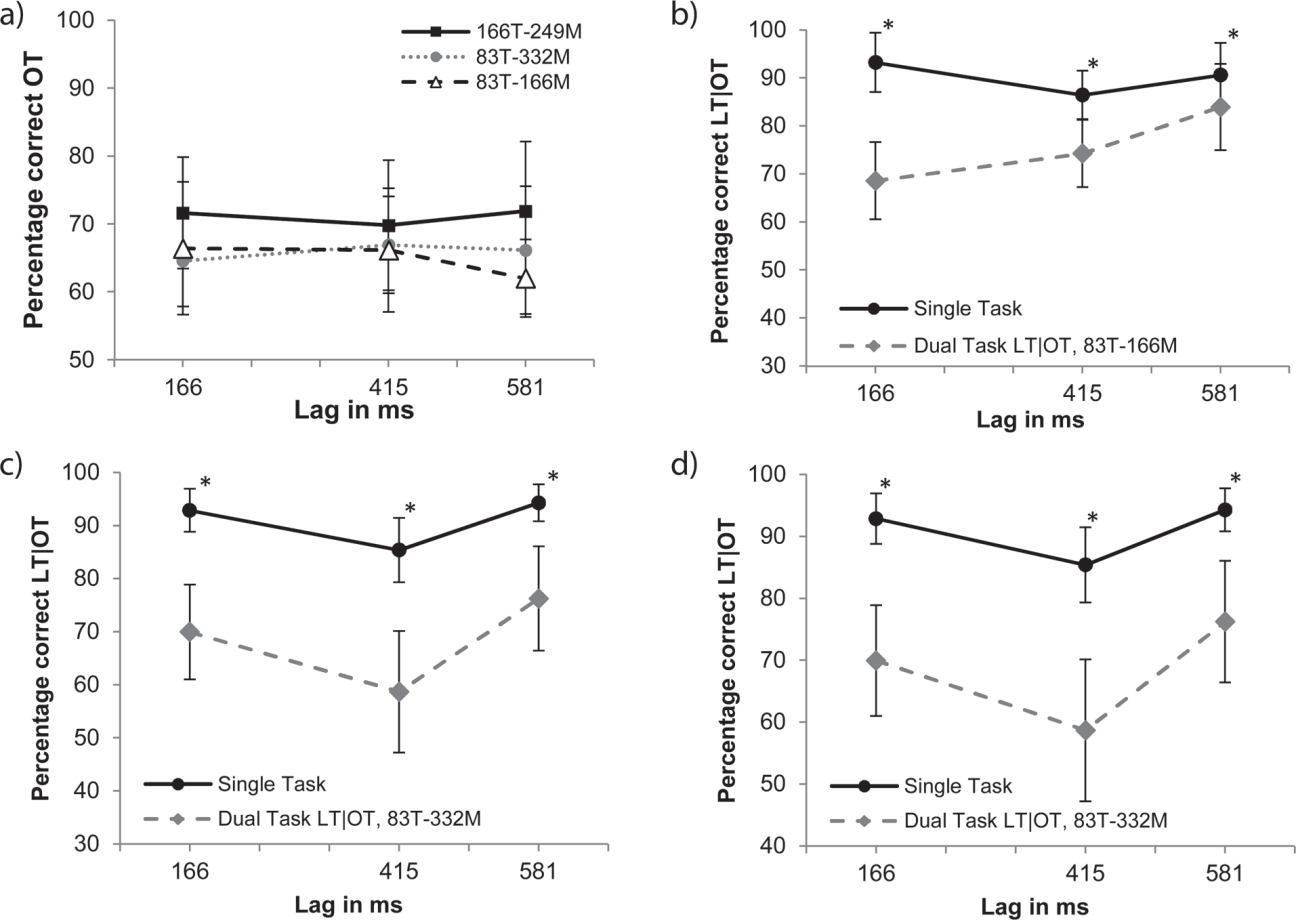
Results of Experiment 4. Panel a: Dual task Gabor detection performance (OT) for different conditions. The dotted line represents the target presentation duration of 83 ms combined with a mask presentation duration of 166 ms. The dashed line represents the 166 and 249 ms combination and the solid line represents the 83 and 332 ms combination. Panels b-d: Single- and dual-task performance given correct OT for letter identification (LT) for the target/mask durations of 83/166 ms, 166/249 ms and 83/332 ms, respectively. Solid lines represent the single-task conditions and dashed lines the dual-task conditions. Stars mark significant differences between single- and dual-task conditions (*p* < 0.001).

**OT detection.** To examine the orientation-detection data shown in Fig. 6A, a 3x3 ANOVA was performed with target/mask duration (83/166 ms, 166/249 ms, 83/332 ms) and lag (166, 415, 581 ms) as factors. An effect for target/mask duration was found [*F*(2,30) = 6.9, *MSE* = 62.2, *p* < 0.003]. Averaged over lags, performance was 64.8% (*SD* = 15.4), 69.7% (*SD* = 20.1) and 64.2% (*SD* = 17.5) for the 83/166 ms, 166/249 ms, and 83/332 ms combinations, respectively. Three Bonferroni-corrected paired-samples t-tests (α = 0.017) compared the OT performance averaged over lags for each target/mask combination. The 166/249 ms combination was shown to have a slight, but significantly higher average compared to the 83/332 ms combination [*t*(15) = 3.64, *p* < 0.002]. This might be explained by the longer target presentation duration in the 166/249 ms condition.

**LT identification.** The LT identification results are shown in Fig. 6B-D. LT-identification data were analyzed with a 3x2x3 ANOVA with target/mask combination (83/166 ms, 166/249 ms and 83/332 ms), condition (single/dual task), and lags (166, 455 and 581 ms) as factors. Effects for condition [*F*(1,15) = 79.8, *MSE* = 292.7, *p* < 0.001], target/mask combination [*F*(2,30) = 5.53, *MSE* = 78.2, *p* < 0.008] and lag [*F*(2,30) = 19.2, *MSE* = 199.5, *p* < 0.001] were found. Interactions also occurred between condition and lag [*F*(2,30) = 8.2, *MSE* = 114.8, *p* < 0.001], target/mask combination and lag [*F*(4,60) = 3, *MSE* = 68.3, *p* < 0.026] and target/mask combination, condition and lag [*F*(4,60) = 2.8, *MSE* = 69, *p* < 0.033]. Performance for dual-task LT identification for the 83/166 ms condition averaged over lags was 92% (*SD* = 7.1) correct in the single- and 77% (*SD* = 9.1) in the dual-task condition (see Panel b). In the 166/249 ms condition performance averaged at 90.3% (*SD* = 7.1) for the single- and 70.6% (*SD* = 12.9) for the dual-task condition (see Panel c). In the 83/332 ms condition, performance averaged at 91% (*SD* = 7.7) and 71.7% (*SD* = 14.3) for the single and dual task respectively (see Panel d).

Nine Bonferroni-corrected paired-samples t-tests (α = 0.006) compared single-task performance of each lag of every target/mask combination with its dual-task counterpart. The single-task condition scored significantly higher (*p* < 0.001) in all cases except at lag 581 ms for the 83/166 ms combination, where performance was found to be comparable for the single- and the dual-task conditions (*p* > 0.98).\

Single-task performance was compared between lags with 3 Bonferroni-corrected (α = 0.006) paired samples t-test within each target/mask combination. Lag 415 ms scores were lower than the other lags, with the exception of the comparison with 581 ms for the 83/166 ms combination.

The dual-task results reveal that interference persists for a longer period when the combined duration of target and mask is increased, irrespective of the target duration. This indicates that the total stimulus duration determines the extend of the interference, consistent with our second hypothesis that the continuous presence of a stimulus impedes disengagement of attention, even when the stimulus itself is no longer task-relevant (e.g. [28,29]).

We also observe lower single-task performance at lag 415 ms compared to other lags for the longer-duration target/mask combinations. This could be due to an exogenous shift of attention away from the letter stream, caused by the offset of the mask. No significant effect was found for the 83/166 ms combination, where the offset occurs at 249 ms, in-between measured latencies.

## General Discussion

In four experiments we examined the time course of dual task interference for a combination of an orientation search task and a letter identification task. In the first experiment, we replicated earlier results revealing clear dual-task interference at nonzero lags when the letter task precedes the search task. This interference is thought not to be due to attentional effects but to competition for higher-level resources, occurring because of an overlap in the processing times of both targets. In the following experiments, we reversed task order, starting out from the hypothesis that when the (relatively easy) search task is performed first, processing resources should be promptly available for the second task. Another effect that should support dual task performance for this presentation order is that the onset of the OT provides a strong endogenous cue for the location of the upcoming T2. In the original presentation order, spatial uncertainty for T2 is elevated, because the orientation target can appear at any location on a radius around fixation [37–41].

Surprisingly, results in Experiment 2 show that dual-task interference was even stronger than in Experiment 1. To examine why this is the case we looked at two alternative hypotheses. In Experiment 3, we explored the hypothesis that the interference is caused by a spatial asymmetry in directing the attentional window. Previous studies have demonstrated that looming stimuli capture attention more readily than receding stimuli do, indicating that performance benefits should occur when the attentional window is expanding, compared to constricting [21–24]. We tested this hypothesis by comparing performance for two radii of the search display. When the refocussing of the attentional window is the factor causing the dual-task interference, performance for the larger radius should be worse than for the smaller radius. However, results did not show any difference between the two conditions and thus we found no support for this first hypothesis [42]. In Experiment 4, we examined our second hypothesis that the dual task interference is caused by difficulties in disengaging attention from the OT search display and ensuing mask while they are onscreen, hampering a switch to the RSVP stream. We combined different target- and mask-durations allowing us to separate effects of target duration from effects of total stimulus duration. The results were clear-cut, in that they only showed a dependence on total stimulus duration, but not on target duration.

Thus, returning to the question posed in the introduction: why is the dual-task interference in the second experiment larger than that found for the original task order in Experiment 1 [3,12]? Our results suggest that the interference, and also several other patterns we observe in our data, are due to the dynamics of visual attention in situations where multiple visual objects are presented. Shifts of attention can be initiated endogenously or exogenously, which means that they are influenced by task goals on the one hand, and by object salience on the other hand (for a comprehensive review, see [43]). Importantly, before a shift and engagement to a new object can occur, attention needs to be disengaged from the current object [13,27].

Evidence for exogenous shifts of attention is provided by our single-task data. In Experiment 2, a clear performance decrease is seen at lag 0 ms, which cannot be not observed in Experiment 1. While the stimuli are exactly the same, in Experiment 2 the single task comprises identification of a target letter, while the orientation target is task-irrelevant, whereas in Experiment 1 this is reversed. Given that the orientation-search display has a larger surface area, a longer presentation duration and contains more elements than the RSVP-stream during target presentation, our results are consistent with an exogenous shift of attention away from the target location towards the onset of an irrelevant but more salient object [13,32–35,44,45]. Further evidence for such attentional capture is provided by the single-task results of Experiment 4. When search- and mask- display have a combined duration of 415 ms, LT identification shows a significant drop at the coinciding lag in the single-task condition, indicating that the offset of the search display mask draws attention away from the central RSVP-stream [46,47]. In contrast, comparison across conditions for lag 166 ms reveals that single-task performance is independent of whether or not LT onset and OT offset coincide. Probably the change from search- to mask-display, which was equiluminant, was not salient enough to draw attention away exogenously.

The larger than expected interference occurring in the dual-task conditions of Experiments 2–4 can be explained by a combination of exogenous an endogenous attentional effects. While the paradigm used in these experiments requires subjects to endogenously shift their attention from the search display to the RSVP stream, the (salient) search display’s mask seems to hold attention exogenously until it offsets. As a result, LT identification suffers at short lags and will only approach single-task performance some time after the masks offset. Support for this explanation is provided by the differences between single- and dual-task performance found across experiments. It appears that the longest lag at which this difference is significant increases monotonically as a function of the combined target/mask duration of the search display: it is 415 ms for the 249-ms duration, 498 ms for the 332-ms duration, and 581 ms for the 415-ms duration. Thus, dual-task performance is still impaired about 150 ms after the mask’s offset, but returns to single-task performance levels at longer lags. While these data already indicate that dual-task performance depends on OT plus mask duration rather than just OT duration, the direct test of this in Experiment 4 provides a more rigorous confirmation.

Object salience not only seems to be the dominant factor explaining the single- and dual-task results of Experiments 2–4, it is also apparent in the results for the original task order of Experiment 1 and in the experiments reported by Ettwig and Bronkhorst in 2013 [12]. The dual-task LT identification data show in most cases a drop at lag 0 ms, which is consistent with an exogenous shift of attention towards the search display. Furthermore, the fact that no dual-task interference is found at nonzero lags for the search displays that involved motion- or color-search indicates that attention can be shifted away very quickly from the RSVP stream to the search display.

As discussed in the Introduction, difficulties in disengaging attention can explain results of several earlier studies involving visual stimuli presented close in time at different locations. In experiments using the gap paradigm, it was found that the continuous presence of a central fixation prolonged saccades towards a peripheral target, compared to conditions where the fixation was removed [28–30]. Moore and colleagues [48] used subsequent targets in a simple search display and masked the first target either directly after its offset or after the offset of the second target. In the former case, discrimination scores for the second target were lower than in the latter case, indicating that the mask interfered with the switch of attention from first to second target. Also in other studies it has been observed that attentional dwell times and difficulties with disengaging of attention can explain performance deficits occurring when locations of subsequent targets are changed. Visser and colleagues [49], for example, observed that lag-1 sparing in the attentional blink task (the absence of a blink for targets presented directly after each other) does not occur when the targets are presented at different locations, and they attributed this to attentional switch costs. Duncan, Martens and Ward [50] compared single-modality with mixed modality (auditory-visual) dual-target performance and found significant deficits in the former case for lags of up to 500 ms. They argued that, since observers then had to monitor 2 separate streams of stimuli aligned vertically and horizontally, attentional dwell times followed by release of attentional capacity, account for this result.

The novel aspect demonstrated by our results is that the penalties caused by difficulties in disengaging attention from a stimulus can be substantial, persisting for hundreds of milliseconds after target offset, and depend strongly on stimulus combination and their order of presentation. This means that when a performance decrease occurs for targets presented at different locations within a short period of time, it is actually not clear to which degree this is due to attentional dwell times and/ or switch costs. For example, given that location changes also occurred in the single-stream control conditions of the Duncan et al. study (targets could be presented left or right of fixation) [50], one would also have expected a performance reduction to occur then. In other words, the effect of the position change on the identification of the second target is small in the single-stream case, it is unclear why it should account for the large performance deficit in the dual-stream condition. Another example illustrating the difficulty in interpreting dual-task results is provided by the study of Arnell and Duncan [20]. Conditions included one in which two subsequent visual targets were presented at randomly changing locations. The authors mainly attributed the large performance deficit occurring then, lasting up to several hundreds of milliseconds, to resource competition at higher processing levels, and this may well be true. The explanation might, however, also be that the deficit is entirely due to the combination of dwell time (on target plus mask) and inefficient switching of attention due to uncertainty of target position.

Our results, therefore, make it clear that dual-task conditions need to be explored in more detail, for example by varying target-interferer intervals, mask durations, or (when applicable) target order, before inferences can be made about causes of dual-task interference.
